# Mouse skeletal muscle adaptations to different durations of treadmill exercise after the cessation of FOLFOX chemotherapy

**DOI:** 10.3389/fphys.2023.1283674

**Published:** 2023-11-01

**Authors:** Jessica L. Halle, Brittany R. Counts, Quan Zhang, Kylie M. James, Melissa J. Puppa, Stephen E. Alway, James A. Carson

**Affiliations:** ^1^ Integrative Muscle Biology Laboratory, Division of Regenerative and Rehabilitative Sciences, University of Tennessee Health Science Center, Memphis, TN, United States; ^2^ The University of Memphis, College of Health Sciences, Memphis, TN, United States; ^3^ Laboratory of Muscle Biology and Sarcopenia, Department of Physical Therapy, University of Tennessee Health Science Center, Memphis, TN, United States

**Keywords:** myokines, exerkines, IL-6, LIF, myostatin, COXIV, cancer survivors

## Abstract

FOLFOX (5-fluorouracil, leucovorin, oxaliplatin) chemotherapy is a treatment for colorectal cancer that can induce persistent fatigue and metabolic dysfunction. Regular exercise after chemotherapy cessation is widely recommended for cancer patients and has been shown to improve fatigue resistance in mice. However, gaps remain in understanding whether the early systemic and skeletal muscle adaptations to regular exercise are altered by prior FOLFOX chemotherapy treatment. Furthermore, the effects of exercise duration on early metabolic and skeletal muscle transcriptional adaptations are not fully established.

**Purpose**: Investigate the effects of prior FOLFOX chemotherapy treatment on the early adaptations to repeated short- or long-duration treadmill exercise, including the fasting regulation of circulating metabolic regulators, skeletal muscle COXIV activity and myokine/exerkine gene expression in male mice.

**Methods**: Male C57BL6/J mice completed 4 cycles of FOLFOX or PBS and were allowed to recover for 4-weeks. Subsets of mice performed 14 sessions (6 d/wk, 18 m/min, 5% grade) of short- (10 min/d) or long-duration (55 min/d) treadmill exercise. Blood plasma and muscle tissues were collected 48–72 h after the last exercise bout for biochemical analyses.

**Results**: Long-duration exercise increased fasting plasma osteocalcin, LIF, and IL-6 in healthy PBS mice, and these changes were ablated by prior FOLFOX treatment. Slow-oxidative soleus muscle COXIV activity increased in response to long-duration exercise in PBS mice, which was blocked by prior FOLFOX treatment. Fast-glycolytic plantaris muscle COXIV activity increased with short-duration exercise independent of FOLFOX administration. There was a main effect for long-duration exercise to increase fasting muscle IL-6 and COXIV mRNA expression independent of FOLFOX. FOLFOX administration reduced muscle IL-6, LIF, and BDNF mRNA expression irrespective of long-duration exercise. Interestingly, short-duration exercise suppressed the FOLXOX induction of muscle myostatin mRNA expression.

**Conclusion:** FOLFOX attenuated early exercise adaptations related to fasting circulating osteocalcin, LIF, and IL-6. However, prior FOLFOX treatment did not alter the exercise adaptations of plantaris muscle COXIV activity and plasma adiponectin. An improved understanding of mechanisms underlying exercise adaptations after chemotherapy will provide the basis for successfully treating fatigue and metabolic dysfunction in cancer survivors.

## 1 Introduction

Regular physical activity promotes a wide range of physiological adaptations that reflect the body’s ability to adjust and respond to the metabolic demands of exercise (Holloszy and Coyle, 1984; McGee and Hargreaves, 2020; Smith et al., 2023). Regular aerobic exercise induces genetic and metabolic adaptations in tissues throughout the body to improve cardiovascular health, mitochondrial function, endurance capacity, and metabolic flexibility (Green et al., 1991; Hellsten and Nyberg, 2011; Drake et al., 2016; Goodpaster and Sparks, 2017). Consequently, exercise is widely recommended for cancer survivors to combat disease, treatment-associated fatigue, and metabolic dysfunction. Contracting skeletal muscle promotes the transcription of various genes involved in exercise adaptations and can release myokines/exerkines to regulate adaptations in other tissues (Pedersen et al., 2007; Keller et al., 2011). Adaptations to regular exercise are differentially affected by exercise dose, which refers to the frequency, intensity, time (duration), and type of exercise performed. Adaptations may be acute, occurring immediately after exercise, or chronic, requiring weeks to months of regular exercise (Egan et al., 2013; Petriz et al., 2017; Hughes et al., 2018; Farrell and Turgeon, 2021). While many studies have described acute exercise responses and adaptations to chronic exercise at different exercise intensities, it is not well established how altering the duration of aerobic exercise sessions may differentially affect early systemic and skeletal muscle adaptations, including muscle myokine/exerkine gene transcription.

Systemic metabolic adaptations to exercise require crosstalk between different tissues and organs, promoting the synthesis and release of numerous circulating cytokines, growth factors, and hormone regulators (Sabaratnam et al., 2022). Skeletal muscle tissue exerts endocrine functions to mediate systemic training adaptations (Severinsen and Pedersen, 2020). Myokines or exerkines released from contracting skeletal muscle include interleukin-6 (IL-6), IL-15, leukemia inhibitory factor (LIF), brain-derived neurotrophic factor (BDNF), and myostatin, which can modulate insulin sensitivity, inflammation, lipid metabolism, and mitochondrial biogenesis across different tissues (Pedersen et al., 2007; Lee and Jun, 2019; Balakrishnan and Thurmond, 2022). Exercise can also promote the release of factors (i.e., exerkines) from adipose tissue and bone, including adiponectin and osteocalcin, to regulate systemic energy metabolism (Mera et al., 2016; Becic et al., 2018). Furthermore, while cancer can disrupt systemic metabolism and skeletal muscle responses to exercise and contraction (Puppa et al., 2014; VanderVeen et al., 2020; Leal et al., 2021), the potential effects of prior treatment with chemotherapy on metabolic responses to regular exercise are not well established.

Regular exercise offers a wide range of benefits across the cancer care continuum. Increased physical activity can alleviate treatment-associated side effects from diagnosis to survivorship and improve patient quality of life (Van Moll et al., 2016; Kirkham et al., 2018; Coletta et al., 2022). FOLFOX (5-fluorouracil [5-FU], leucovorin, oxaliplatin) chemotherapy is a regimen used to treat colorectal cancer that can cause long-lasting metabolic dysfunction, skeletal muscle mitochondria damage, fatigue and weakness in clinical and preclinical models (Denlinger and Barsevick, 2009; Barreto et al., 2016; Chung et al., 2020; Halle et al., 2023). We previously demonstrated that short-duration treadmill exercise performed regularly over two-weeks improves fatigue resistance in mice after the cessation of FOLFOX chemotherapy (Halle et al., 2022). However, it is unclear if prior treatment with FOLFOX alters the fasting regulation of circulating metabolic regulators and skeletal muscle myokine/exerkine-related exercise adaptations. Understanding the impact of chemotherapy on exercise adaptations is essential for prescribing feasible training programs to cancer patients who may encounter unique challenges and physical limitations.

The beneficial health effects of acute exercise on systemic metabolism are well documented, and repeated bouts of exercise can induce systemic and skeletal muscle adaptations that improve metabolic flexibility (Thyfault and Bergouignan, 2020). Health-related adaptations can involve circulating cytokines and growth factors regulating systemic metabolism and energy availability during fasting, feeding, and physical activity, including the IL-6 family of cytokines, adiponectin, and osteocalcin (Mera et al., 2016; Becic et al., 2018; Severinsen and Pedersen, 2020). The time course of exercise-induced adaptations is directly related to the duration, frequency, intensity, and mode of the repeated exercise (Bland et al., 2021). Furthermore, exercise can differentially induce changes in skeletal muscle subtypes that vary in functional and metabolic properties, such as slow-oxidative and fast-glycolytic muscle (Smith et al., 2023). We investigated the effects of prior FOLFOX chemotherapy treatment on the early adaptations to repeated bouts of short- or long-duration treadmill exercise training including the fasting regulation of circulating metabolic regulators, skeletal muscle COXIV activity, and myokine/exerkine gene expression in male mice. We hypothesized that prior FOLFOX treatment would inhibit the early adaptations to repeated treadmill exercise involving circulating regulators of energy metabolism, skeletal muscle oxidative metabolism, and myokine/exerkine gene expression. Early adaptations to repeated exercise sessions were investigated in healthy mice using treadmill exercise performed for either 10 min/d (short) or 55 min/d (long) durations, 6 days a week for 14 total sessions. A subset of mice performed treadmill exercise after completing 4 cycles of FOLFOX chemotherapy, which has previously been shown to induce deficits in physical function and systemic metabolism (Halle et al., 2022; Halle et al., 2023). Mice were sacrificed 48–72 h after the last treadmill session to limit the acute effects of the last exercise bout. COXIV activity was examined in fast-glycolytic plantaris and slow-oxidative soleus muscles to investigate the impact of repeated short- and long-duration exercise sessions on skeletal muscle oxidative metabolism. Mice were fasted for 12-hours to examine the effect of exercise on circulating metabolic regulators and muscle myokine/exerkine gene expression.

## 2 Materials and methods

### 2.1 Animals

Male C57BL/6J mice were purchased from The Jackson Laboratory at 10-week of age and were allowed to acclimate to the facilities for 2-week. Mice were group housed (5 mice per cage) and kept on a 12:12-h light/dark cycle beginning at 0600 h. Mice were fed standard rodent chow *ad libitum* (Harlan Teklad Rodent Diet #7912). Before euthanasia, mice were fasted overnight and euthanized at the start of the light cycle. At the time of tissue collection, mice were anesthetized (3%–5% isoflurane) via inhalation (SomnoSuite, Kent Scientific); anesthetic depth was confirmed by toe-pinch assessment, and mice underwent cervical dislocation prior to tissue collection. All experiments were approved by the University of Tennessee Health Science Center Animal Care and Use Committee.

### 2.2 Chemotherapy treatment and recovery

At 12–13 weeks of age, mice were randomized into treatment groups and administered i. p. injections of either phosphate-buffered saline (PBS) or FOLFOX chemotherapy (5-Fluorouracil [5-FU]: 30 mg/kg BW, Oxaliplatin: 6 mg/kg BW, Leucovorin: 90 mg/kg BW [Sigma #F8423, #O9512, #PHR1541]) as described (Halle et al., 2023). One cycle of chemotherapy consisted of an injection followed by 2-week of monitoring, and four cycles were completed. Following the fourth injection, mice were allowed to recover for a total of 4-week; exercise training was performed in subsets of mice during the last 2-week of recovery, as described below.

### 2.3 Treadmill exercise

**FIGURE 1:**
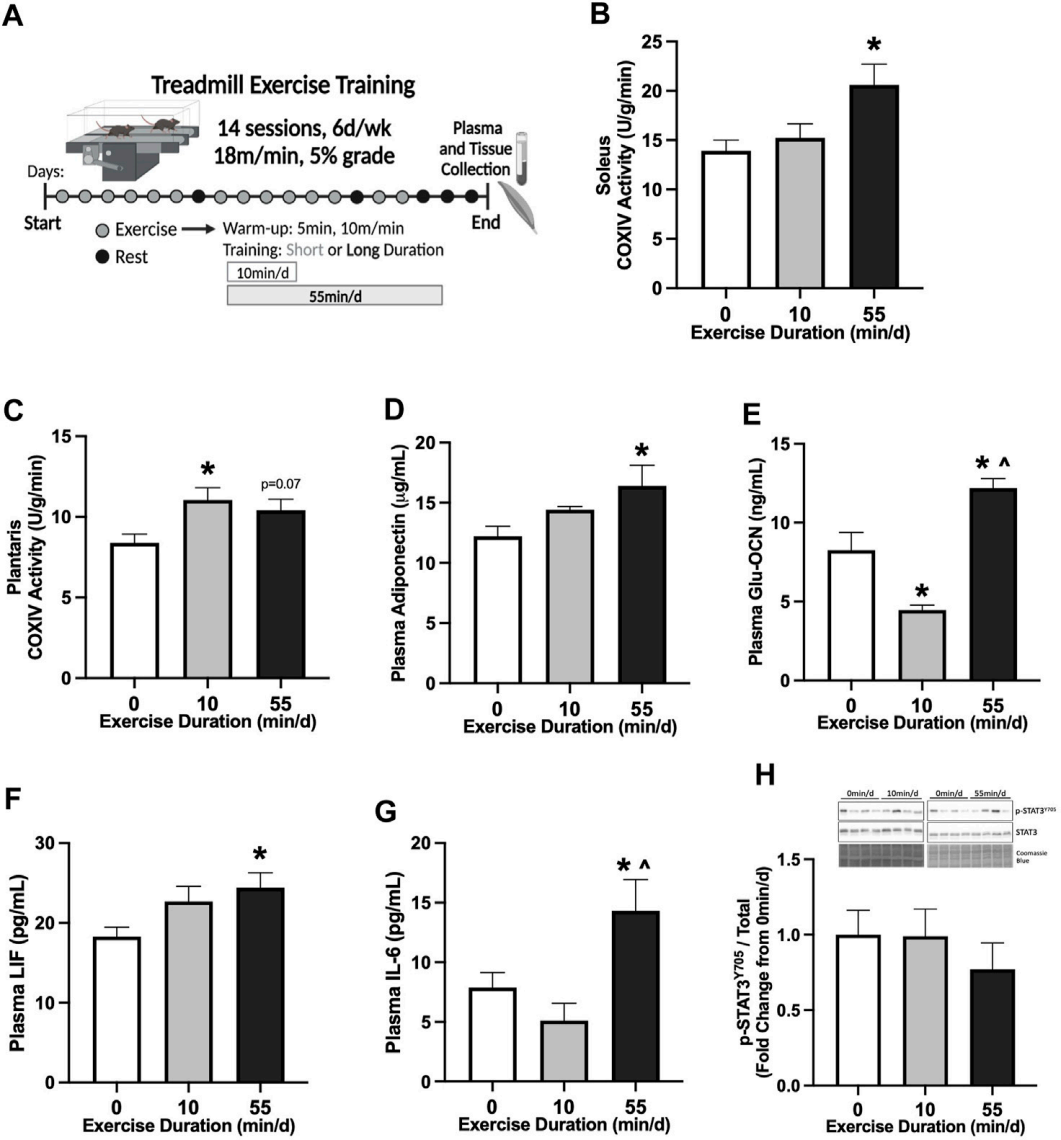
Skeletal muscle and systemic adaptations to 14 sessions of repeated of short- or long-duration treadmill exercise in healthy mice. **(A)** Study Design: Male C57BL/6J mice performed 14 sessions (6 d/wk, 18 m/min, 5% grade) of 0 min/d (sedentary), 10 min/d (short-duration), or 55 min/d (long-duration) treadmill exercise. Blood plasma and muscle tissues were collected 48–72 hrs after the last exercise bout, after a 12-hr fast. **(B)** COXIV enzyme activity in oxidative soleus muscle and **(C)** glycolytic plantaris muscle in healthy PBS mice, 0 min/d: N = 13, 10 min/d: N = 9, and 55 min/d: N = 5 for soleus and N = 10 for plantaris. Fasting plasma concentrations of **(D)** adiponectin, **(E)** uncarboxylated osteocalcin (Glu-OCN), **(F)** leukemia inhibitory factor (LIF), and **(G)** interleukin-6 (IL-6) in healthy PBS mice, 0 min/d: N = 10–13, 10 min/d: N = 8–9 and 55 min/d: N = 10. **(H)** Representative Western blot and quantified phosphorylated (p-) to total STAT3 (Y705) protein expression with Coomassie Blue stain as a protein loading control in healthy PBS mice, 0 min/d: N = 12–13, 10 min/d: N = 9, and 55 min/d: N = 10. Data is presented as mean ± SEM. Data is analyzed using One-Way ANOVA with Tukey’s posthoc. Statistical significance was set to *p* < 0.05. * different from 0 min/d; ^ different from 10 min/d.

Treadmill exercise was performed as described (Halle et al., 2022). Healthy PBS control mice, or mice recovering from FOLFOX chemotherapy began exercise at 20–21 weeks of age. Exercise was performed for 14 total sessions to examine early training adaptations (Figure 1A). Treadmill running sessions were performed at the start of the dark cycle (1800 h) in the dark. Mice were acclimated to the treadmill (Columbus Instruments) 2 days before the first session. Acclimation consisted of running at 5% grade for 5-minutes at 5 meters per minute (m/min), 5 min at 10 m/min, and finally, 5-min at 15 m/min. Mice were encouraged to run by gentle hand prods. Exercise frequency was 6 days per week and totaled 14 sessions (Figure 1A). The exercise dose was manipulated by altering the exercise duration. Each session consisted of a 5-minute warm-up at 10 m/min, 5% grade, after which speed was increased to 18 m/min. Mice ran at this speed for 55 min (long-duration) or 10 min (short-duration) (Figure 1A). Mice ran for a total of 60 min/d or 15 min/d each day, including the warm-up period; figures denote time ran at the target exercise speed. Treadmill speed (18 m/min) and grade (5%) remained constant throughout each session. The acute effects of the exercise were minimized by having all mice euthanized for tissue collection 48–72 h after the last exercise session to limit the acute effects of the last exercise bout. Sedentary 0 min/d: PBS N = 10–15, FOLFOX N = 9–12; Long duration exercise 55 min/d: PBS N = 10, FOLFOX N = 10; Short-duration exercise 10 min/d: PBS N = 9, FOLFOX N = 10.

### 2.4 Tissue collection

Following a 12-hr overnight fast, mice were anesthetized (3%–5% isoflurane) and underwent cervical dislocation. Hindlimb muscles were excised, cleared of excessive connective tissue, weighed, snap-frozen in liquid nitrogen, and stored at −80°C. The gastrocnemius muscle was cut and sectioned into the visually red portion between the medial and lateral gastrocnemius heads as described (Halle et al., 2022). Red gastrocnemius was used for protein and gene expression analyses. Soleus and plantaris muscles were used for the COXIV enzyme activity assay.

### 2.5 Enzyme-linked immunosorbent assays

Blood was collected at sacrifice via a retro-orbital sinus puncture with heparinized capillary tubes at sacrifice following a 12-hr overnight fast. Blood was centrifuged at 10,000 × g for 10 min at 4°C. Plasma was stored at −80°C until analyses and assays were run with all samples available. Plasma uncarboxylated osteocalcin (Glu-OCN) (Biomatik #EKF57818), leukemia inhibitory factor (LIF) (Biomatik, #EKU11866), IL-6 (R&D systems #M6000B-1), and adiponectin (R&D Systems #MRP300) concentrations were determined using commercially available ELISA kits according to the manufacturer’s instructions.

### 2.6 Cytochrome c oxidase (COXIV) enzyme activity

Cytochrome c oxidase (COXIV) enzyme activity was measured as described (Halle et al., 2023). Briefly, soleus and plantaris muscles were homogenized by hand using a PYREX^TM^ glass pestle tissue grinder in extraction buffer (0.1M KH2PO4/Na2HPO4, 2 mM EDTA, pH 7.2) at 1:20 mass/volume. Bradford assay was used to determine protein concentration; 20 µg of muscle protein was used for the assay. COXIV colorimetric enzyme activity assay measured the oxidation rate of reduced cytochrome c at 550 nm (BioVision #K287).

### 2.7 RNA isolation, cDNA synthesis, and real-time PCR

RNA isolation, cDNA synthesis, and real-time PCR (RT-PCR) were performed as described (Halle et al., 2023). Total RNA was isolated from 15 to 20 mg of frozen red gastrocnemius tissue sections using TRIzol reagent (Invitrogen #15596018) per manufacturer’s guidelines. After phenol-chloroform extraction, RNA was purified using the PureLink^®^ RNA Mini Kit (Invitrogen #12183018) and eluted in nuclease-free water. Total RNA concentration and purity were measured using spectrophotometry. cDNA was reverse transcribed from 1 μg of total RNA using Superscript IV Reverse Transcriptase and random hexamers according to manufacturer guidelines (Invitrogen #18090200). RT-PCR was performed in 10 μL reactions using 2XPowerTrack SYBR Green master mix, 2 μL of cDNA, 0.5 μL of forward and reverse primers (500 nM), and nuclease-free water. Forward and reverse primer sequences (Supplementary Table S1) were synthesized by Integrated DNA Technologies (IDT) and validated by agarose gel electrophoresis. Melt-curve analysis ensured that the PCR reaction produced only one amplicon. RT-PCR was carried out on the Applied Biosystems QuantStudio3 system. Reactions were incubated for 2-min at 95°C, followed by 40 cycles of a 15-s denaturation step at 95°C, and a 1-min annealing/extension step at 60°C. The 2^−ΔΔCT^ method was used to determine gene expression changes between treatment groups with the GAPDH Ct as the correction factor.

### 2.8 Western blotting

Western blot analysis was performed as described (Halle et al., 2023). Briefly, 15–20 mg frozen red gastrocnemius muscle sections were homogenized in Mueller buffer and protein concentrations were determined using the Bradford method. Muscle homogenates were fractionated on 10% SDS-polyacrylamide gels and transferred to PVDF membranes. Ponceau S (Fisher #501037137) stain was used after protein transfer to ensure equal loading of each gel. Membranes were washed in Tris-buffered saline with 0.1% Tween-20 (TBST) and blocked for 1-hr in 5% milk-TBST at room temperature. Primary antibodies were purchased from Cell Signaling Technology for p-STAT3 (Y705) (#9145) and STAT3 (#4904). Primary antibodies were incubated overnight in 5% milk-TBST diluted 1:1000 or 1:2000, respectively. Membranes were washed and incubated in 5% milk-TBST plus anti-rabbit (#7074) IgG horseradish-peroxidase conjugated secondary antibody diluted 1:4000 for 1-hr at room temperature. Enhanced chemiluminescence (Prometheus Pro-Signal Femto, Genesee Scientific #20–302) was used to visualize antibody-antigen interactions. Immunoblot images were digitally imaged using the iBright 1500 system (Invitrogen), and blots were quantified by densitometry using imaging software (ImageJ, NIH). After imaging, membranes were washed and stained with Coomassie Blue protein stain.

### 2.9 Statistical analysis

Results are reported as mean ± SEM. One-way ANOVA analyses with Tukey’s *post hoc* test were used to examine differences across sedentary (0 min/d), short- (10 min/d), and long-duration (55 min/d) exercise groups in healthy mice or in mice recovering from FOLFOX. Student’s unpaired t-tests were used to compare baseline differences between PBS and FOLFOX sedentary (0 min/d) groups for systemic and skeletal muscle outcomes. Two-way ANOVA analyses were used to compare across treatment (PBS, FOLFOX) and exercise (0 min/d and 55 min/d, or 0 min/d and 10 min/d) for skeletal muscle gene expression. 2 × 3 ANOVA analyses were used to examine across exercise groups (0 min/d, 10 min/d, 55 min/d) and treatment groups (PBS, FOLFOX) and can be found in Supplementary Figures S1, S2. Tukey’s *post hoc* analysis was used when a significant interaction was present. Pearson r correlation coefficients were used to determine associations. Each table and figure legend indicates specific statistical analyses and group N’s. Statistical analysis and figures were prepared using GraphPad (Prism 8 for Mac OS X). Statistical significance was set to *p* < 0.05 for all measures.

## 3 Results

### 3.1 Skeletal muscle and systemic adaptations to 14 sessions of repeated short- or long-duration treadmill exercise in healthy mice

We first examined the early adaptations to 14 sessions of treadmill exercise in healthy male B6 mice (20–21 week of age). The effect of exercise session duration was examined with repeated bouts of either short- (10 min/d) or long-duration (55 min/d) moderate-intensity (18 m/min, 5% grade) exercise. Treadmill exercise bouts were repeated for 14 sessions (6 d/wk) (Figure 1A). Therefore, over the 14 sessions, the short-duration group exercised for 140 min, while the long-duration exercise mice completed 770 min of moderate-intensity treadmill exercise. Plasma and tissue collections were completed in all treatment groups after an overnight 12-hr fast, 48–72-hrs after the last exercise session. Cytochrome c oxidase subunit 4 (COXIV) enzyme activity was measured in slow-oxidative (soleus) and fast-glycolytic (plantaris) skeletal muscle samples from sedentary (0 min/d) and exercised mice to examine early adaptations related to mitochondrial function. Two-weeks of 55 min/d exercise increased soleus muscle COXIV enzyme activity 48% compared to sedentary (0 min/d) (Figure 1B). Short-duration exercise for 10 min/d did not alter soleus muscle COXIV enzyme activity compared to sedentary and long-duration exercise (Figure 1B). There was a differential response to exercise duration in the fast-glycolytic plantaris muscle. There was a nonsignificant trend for long-duration exercise to increase plantaris muscle COXIV activity (Figure 1C). Short-duration exercise increased plantaris muscle COXIV activity by 32% compared to sedentary (Figure 1C). Long-duration exercise sessions for 55 min/d increased plasma adiponectin by 34% compared to sedentary (Figure 1D). Short-duration exercise sessions for 10 min/d did not alter plasma adiponectin compared to sedentary and long-duration exercise (Figure 1D). Long-duration exercise increased plasma uncarboxylated osteocalcin (Glu-OCN) by 48% compared to sedentary and 173% compared to short-duration exercise (Figure 1E). Short-duration exercise reduced plasma Glu-OCN -46% compared to sedentary (Figure 1E). Long-duration exercise increased plasma LIF by 33% compared to sedentary (Figure 1F). Short-duration exercise did not alter plasma LIF (Figure 1F). Long-duration exercise increased plasma IL-6 by 82% compared to sedentary and 180% compared to short-duration exercise (Figure 1G). Short-duration exercise did not alter plasma IL-6 compared to sedentary (Figure 1G). Red gastrocnemius skeletal muscle phosphorylated to total STAT3 (Y705) protein expression was examined to assess the effect of exercise on downstream skeletal muscle cytokine signaling. There were no differences between sedentary, short-, and long-duration exercise for muscle STAT3 phosphorylation in healthy PBS mice (Figure 1H). These data demonstrate that 14 sessions of repeated treadmill exercise was sufficient to induce skeletal muscle and systemic adaptations in a dose-dependent manner. Specifically, long-duration exercise increased soleus muscle COXIV enzyme activity, while short-duration exercise increased plantaris COXIV enzyme activity. Long-duration exercise was required for systemic adaptations related to fasting plasma adiponectin, Glu-OCN, LIF, and IL-6.

### 3.2 Effect of prior FOLFOX chemotherapy treatment on muscle and systemic adaptations to 14 sessions of repeated short- or long-duration treadmill exercise

**FIGURE 2:**
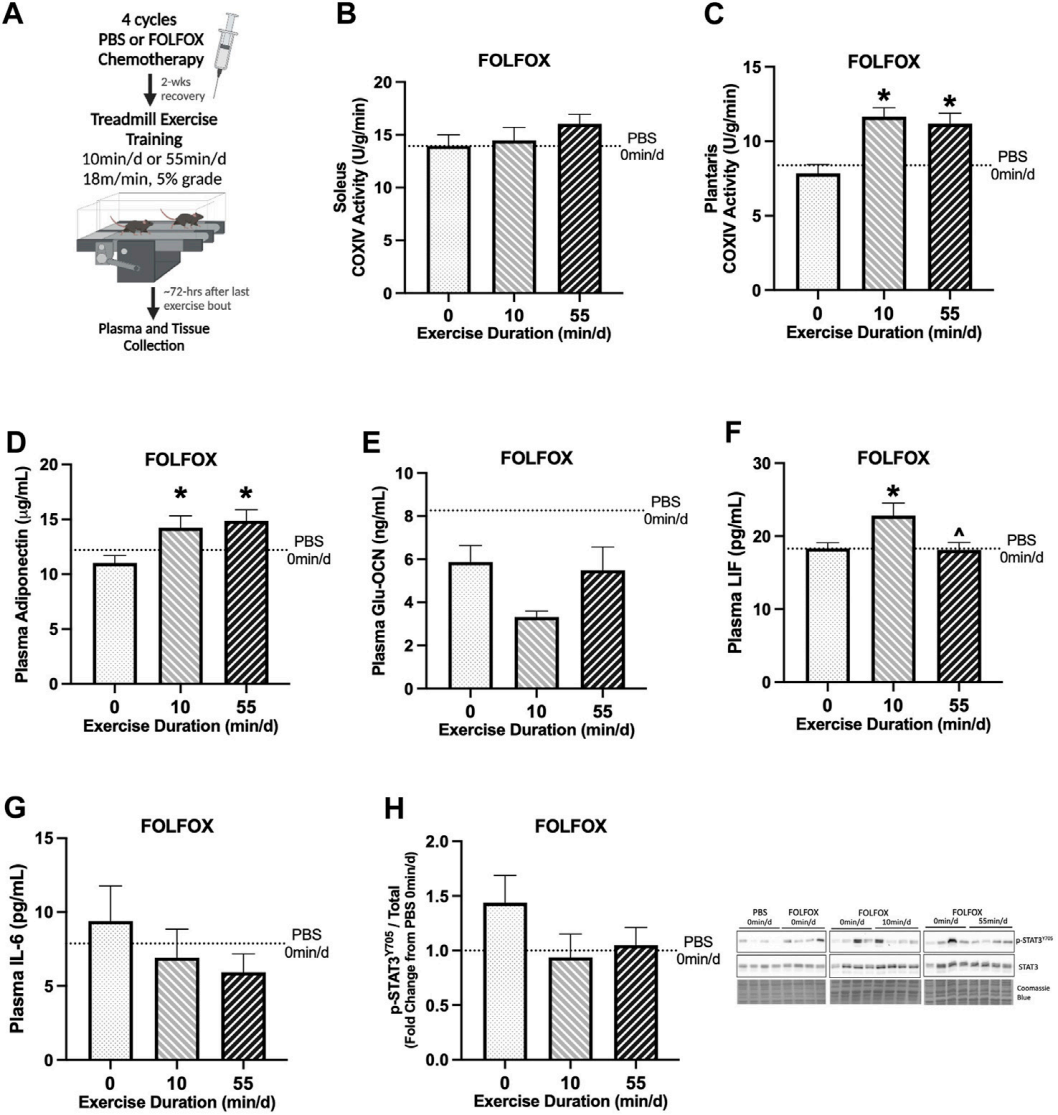
Effect of prior FOLFOX chemotherapy treatment on muscle and systemic adaptations to 14 sessions of repeated short- or long-duration treadmill exercise. **(A)** Study Design: Male C57BL/6J mice were injected with 4 cycles (1 cycle = 1 injection every other wk) of FOLFOX (5-Fluorouracil 30 mg/kg, Oxaliplatin 6 mg/kg, Leucovorin 90 mg/kg) and were allowed to recover for 2-weeks after the last injection. Subsets of mice performed 14 sessions of (6 d/wk, 18 m/min, 5% grade) of 0 min/d (sedentary), 10 min/d (short-duration), or 55 min/d (long-duration) treadmill exercise. Blood plasma and muscle tissues were collected 48–72 hrs after the last exercise bout, after a 12-hr fast. **(B)** Soleus muscle and **(C)** plantaris muscle COXIV enzyme activity in sedentary and exercise trained mice recovering from FOLFOX chemotherapy; 0 min/d (N = 9–12), 10 min/d (N = 9–10) and 55 min/d (N = 10). Fasting plasma concentrations of **(D)** adiponectin, **(E)** uncarboxylated osteocalcin (Glu-OCN), **(F)** leukemia inhibitory factor (LIF), and **(G)** interleukin-6 (IL-6) in FOLFOX mice; 0 min/d: N = 9–12, 10 min/d: N = 9–10, and 55 min/d: N = 10. **(H)** Representative Western blot and quantified phosphorylated (p-) to total STAT3 (Y705) protein expression with Coomassie Blue stain as a protein loading control in mice recovering from FOLFOX; 0 min/d: N = 12, 10 min/d: N = 10, and 55 min/d: N = 10. Data is presented as mean ± SEM. Data is analyzed using One-Way ANOVA with Tukey’s posthoc. Statistical significance was set to *p* < 0.05. * different from 0 min/d; ^ different from 10 min/d.

The effects of prior FOLFOX chemotherapy treatment on the early adaptations to repeated bouts of treadmill exercise were examined (Figure 2A). First, baseline differences between PBS and FOLFOX sedentary (0 min/d) groups were examined using an unpaired t-test. We analyzed COXIV enzyme activity in a subset of control mice, as previously described (Halle et al., 2023), to examine the effects of exercise during recovery from FOLFOX chemotherapy. There were no differences between sedentary PBS and sedentary FOLFOX treatments for soleus and plantaris muscle COXIV enzyme activity (Halle et al., 2023). There were no differences between PBS and FOLFOX treatments for plasma adiponectin (*p* = 0.287) (Figure 2D). There was a trend for sedentary FOLFOX-treated mice to have lower plasma Glu-OCN than sedentary PBS-treated mice (*p* = 0.082) (Figure 2E). There were no differences between PBS and FOLFOX treatments for plasma LIF (*p* = 0.980) or plasma IL-6 (*p* = 0.580) (Figures 2F,G). Red gastrocnemius skeletal muscle phosphorylated to total STAT3 (Y705) protein expression was not different between sedentary PBS and FOLFOX-treated mice (*p* = 0.155) (Figure 2H).

Next, we examined the impact of prior FOLFOX chemotherapy treatment on adaptations to repeated short- and long-duration bouts of exercise. There were no differences between sedentary, short-, and long-duration exercise for slow-oxidative soleus muscle COXIV activity (Figure 2B). Long-duration exercise increased plantaris muscle COXIV activity by 43% compared to sedentary FOLFOX-treated mice (Figure 2C). Short-duration exercise increased plantaris muscle COXIV activity by 49% compared to sedentary FOLFOX-treated mice (Figure 2C). Long-duration exercise increased plasma adiponectin by 35%, and short-duration exercise increased plasma adiponectin by 29% compared to sedentary (Figure 2D). There were no differences between sedentary, short-, and long-duration exercise for plasma Glu-OCN in FOLFOX-treated mice (Figure 2E). There were no differences between sedentary and long-duration exercise for plasma LIF in FOLFOX-treated mice (Figure 2F). Short-duration exercise increased plasma LIF by 49% compared to sedentary and 26% compared to long-duration exercise (Figure 2F). There were no differences between sedentary, short-, and long-duration exercise for plasma IL-6 in FOLFOX-treated mice (Figure 2G). There were no differences between sedentary, short-, and long-duration exercise for phosphorylated to total STAT3 (Y705) protein expression in FOLFOX-treated mice (Figure 2H). These data demonstrate that prior FOLFOX chemotherapy treatment alters early treadmill exercise adaptations observed in healthy PBS mice. However, prior FOLFOX treatment did not alter the exercise adaptations of plantaris muscle COXIV activity and plasma adiponectin.

### 3.3 Effect of prior FOLFOX chemotherapy treatment on skeletal muscle myokine/exerkine gene expression following repeated long-duration exercise bouts

**FIGURE 3:**
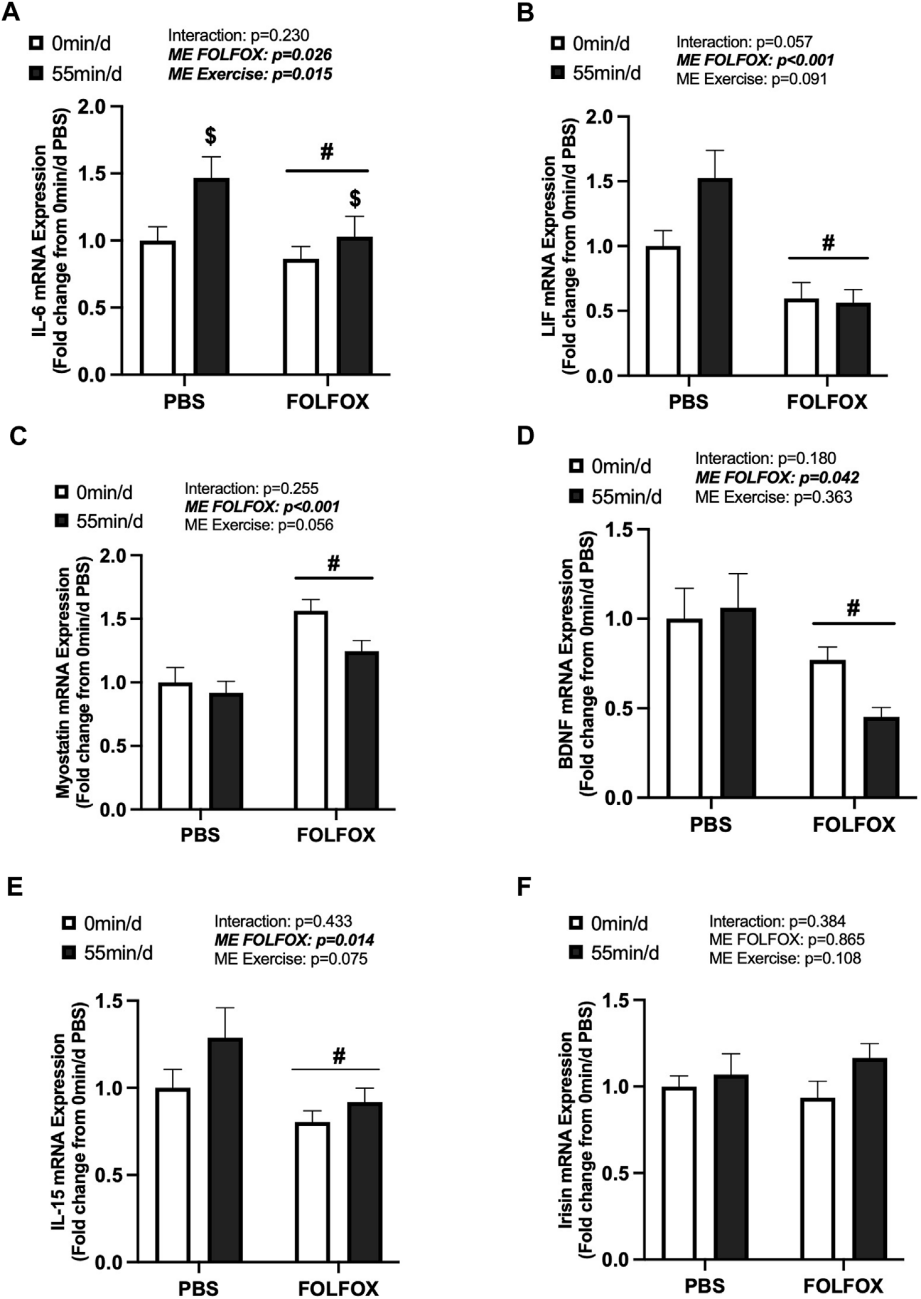
Effects of prior FOLFOX chemotherapy treatment on skeletal muscle myokine/exerkine gene expression following repeated long-duration exercise bouts. Red gastrocnemius expression of **(A)** IL-6, **(B)** LIF, **(C)** myostatin, **(D)** brain-derived neurotrophic factor (BDNF), **(E)** IL-15, and **(F)** irisin mRNA in PBS and FOLFOX sedentary and long-duration exercise trained mice. GAPDH was used as the housekeeping gene. 0 min/d: PBS N = 13, FOLFOX N = 12, 55 min/d: PBS N = 10, FOLFOX N = 10. Data is presented as mean ± SEM. Data is analyzed using Two-Way ANOVA. Statistical significance was set to *p* < 0.05. Bold and italicized values are significant. # main effect of FOLFOX, $ main effect of 55 min/d (long-duration) exercise.

Skeletal muscle myokine/exerkine gene expression was examined in PBS and FOLFOX-treated mice following repeated bouts of long-duration treadmill exercise. There was a main effect of long-duration exercise to increase skeletal muscle IL-6 mRNA expression in PBS and FOLFOX mice (Figure 3A). There were no effects of long-duration exercise to alter muscle LIF, myostatin, BDNF, IL-15, or irisin mRNA expression in PBS and FOLFOX mice (Figures 3B–F). A pre-planned *t-test* was used to examine differences between sedentary and long-duration exercise in PBS-treated mice for red gastrocnemius LIF mRNA expression. Compared to sedentary, long-duration exercise increased muscle LIF mRNA expression in healthy PBS-treated mice (53%; *p* = 0.033) (Figure 3B). There was a main effect for FOLFOX to reduce muscle IL-6, LIF, BDNF, and IL-15 mRNA expression in sedentary and long-duration exercised mice (Figure 3A, B, D, E). There was a main effect for FOLFOX to increase myostatin mRNA expression in sedentary and long-duration exercised mice (Figure 3C). There were no effects of exercise or FOLFOX to alter muscle irisin mRNA expression (Figure 3F). Together, these data show that repeated bouts of long-duration exercise increased IL-6 mRNA expression but did not alter the expression of other myokines/exerkines, including LIF, myostatin, BDNF, IL-15, and irisin. Regardless of exercise, prior treatment with FOLFOX chemotherapy suppressed the mRNA expression of myokines/exerkines, including IL-6, LIF, BDNF, and IL-15 and increased skeletal muscle myostatin mRNA expression.

### 3.4 Effect of FOLFOX on skeletal muscle metabolic-related gene expression following repeated bouts of long-duration treadmill exercise

**FIGURE 4:**
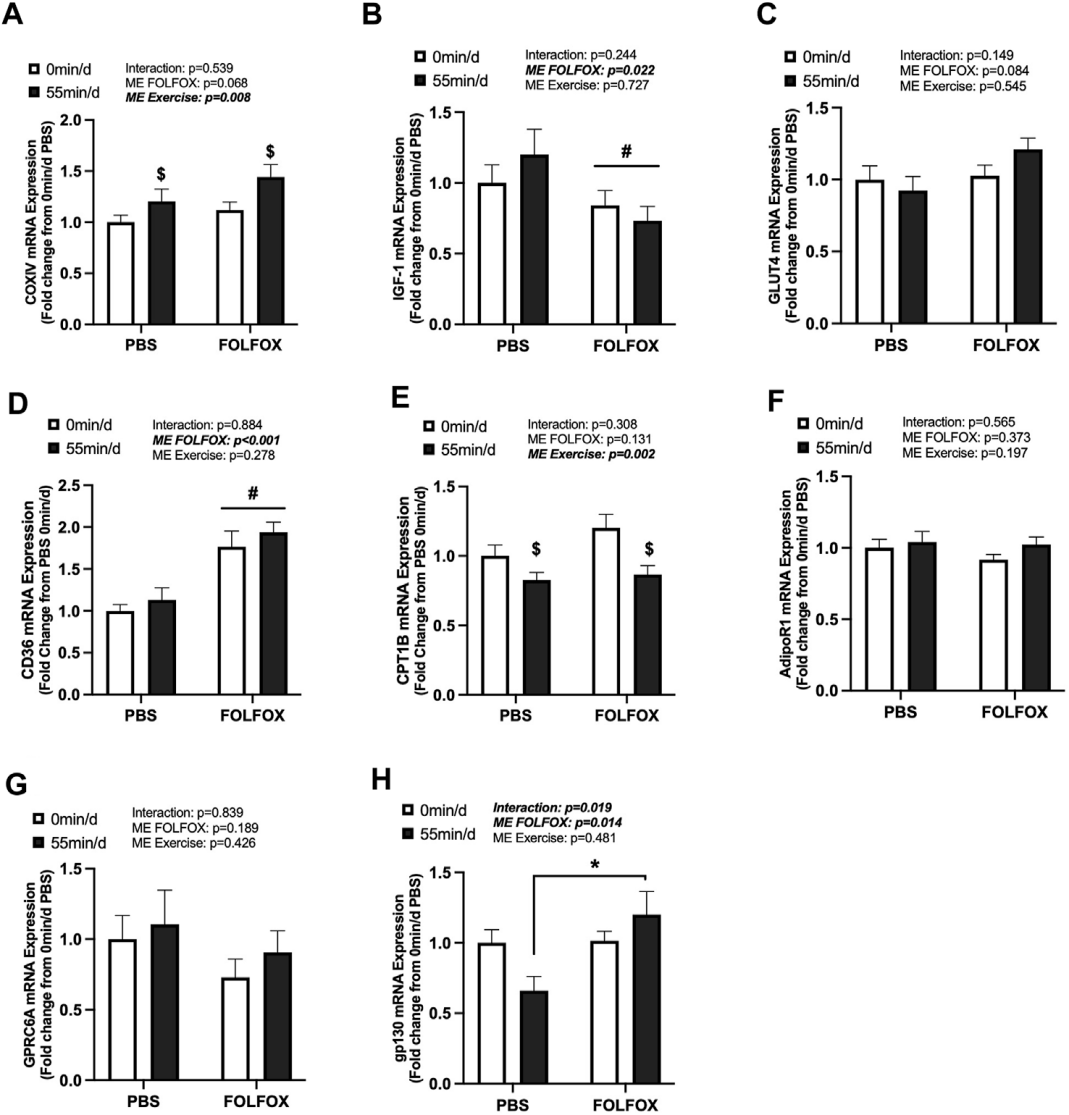
Effect of prior FOLFOX chemotherapy treatment on skeletal muscle metabolic-related gene expression following repeated bouts of long-duration treadmill exercise. Red gastrocnemius expression of **(A)** COXIV, **(B)** insulin-like growth factor 1 (IGF-1), **(C)** glucose transporter type 4 (GLUT4), **(D)** CD36, **(E)** carnitine palmitoyltransferase 1B (CPT1B), **(F)** AdipoR1, **(G)** G protein-coupled receptor class C group 6 member A (GPRC6A), and **(H)** glycoprotein 130 (gp130) mRNA in PBS and FOLFOX sedentary (0 min/d) and long-duration (55 min/d) exercise trained mice. GAPDH was used as the housekeeping gene. (0 min/d): PBS N = 12–13, FOLFOX N = 12, (55 min/d): PBS N = 10, FOLFOX N = 10. Data is presented as mean ± SEM. Data is analyzed using Two-Way ANOVA and Tukey’s *post hoc*. Statistical significance was set to was set to *p* < 0.05. Bold and italicized values are significant. * with bracket denotes significant difference between respective groups. # main effect of FOLFOX, $ main effect of (55 min/d) (long-duration) exercise.

The effects of long-duration exercise and FOLFOX chemotherapy treatment on metabolic-related gene expression were examined. Additionally, we examined the gene expression of skeletal muscle receptors for circulating factors, including adiponectin (AdipoR1), Glu-OCN (GPRC6A), LIF (gp130), and IL-6 (gp130). There was a main effect of long-duration exercise to increase skeletal muscle COXIV mRNA expression in PBS and FOLFOX-treated mice (Figure 4A). There were no effects of long-duration exercise to alter the expression of other metabolic genes, including IGF-1, GLUT4, and CD36 (Figures 4B–D). There was a main effect of long-duration exercise to reduce muscle CPT1B mRNA expression in PBS and FOLFOX-treated mice (Figure 4E). Long-duration exercise for 55 min/d did not alter the mRNA expression of AdipoR1, GPRC6A, and gp130 (Figures 4F–H). There was a main effect for FOLFOX to reduce IGF-1 mRNA expression (Figure 4B) and increase CD36 mRNA expression (Figure 4D) in sedentary and long-duration exercised mice. Gp130 mRNA expression was higher in long-duration exercised FOLFOX mice compared to long-duration exercised PBS mice (Figure 4H). Together, these data demonstrate that 14 sessions of repeated long-duration exercise was sufficient to induce skeletal muscle COXIV mRNA expression; however, other metabolic-related genes were unaffected by long-duration exercise. Interestingly, prior treatment with FOLFOX chemotherapy suppressed IGF-1 mRNA expression regardless of exercise.

### 3.5 The effect of repeated short-duration exercise sessions on skeletal muscle COXIV and myokine/exerkine gene expression in PBS and FOLFOX treated mice

**FIGURE 5:**
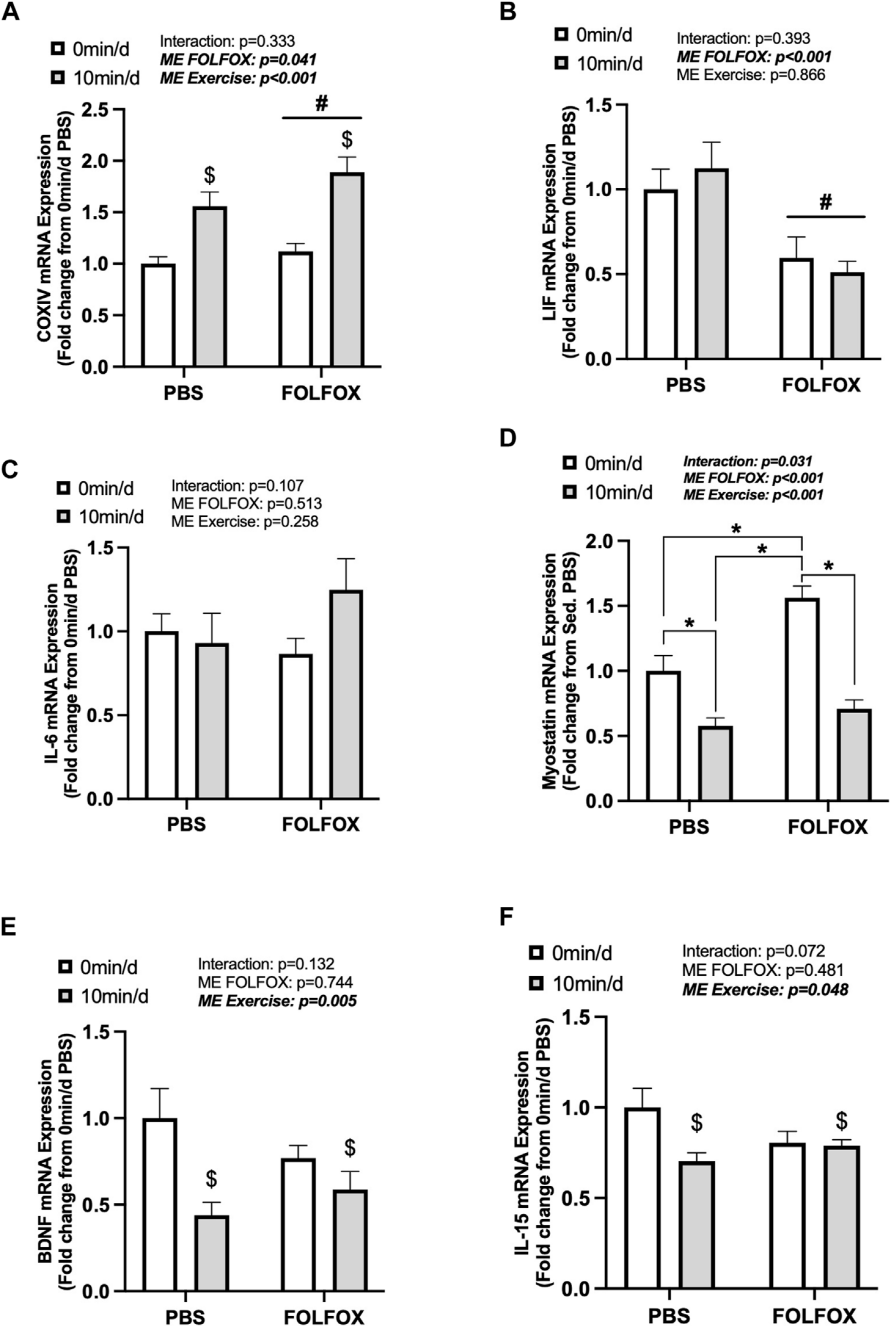
The effect of repeated short-duration treadmill exercise sessions on skeletal muscle COXIV and myokine/exerkine gene expression in FOLFOX treated mice. Red gastrocnemius expression of **(A)** COXIV, **(B)** LIF, **(C)** IL-6, **(D)** myostatin, **(E)** BDNF, and **(F)** IL-15 mRNA expression in PBS and FOLFOX sedentary (0 min/d) and short-duration (10 min/d) exercise trained mice. GAPDH was used as the housekeeping gene. (0 min/d): PBS N = 13, FOLFOX N = 12, (10 min/d): PBS N = 9, FOLFOX N = 10. Data is presented as mean ± SEM. Data is analyzed using Two-Way ANOVA and Tukey’s *post hoc*. Statistical significance was set to was set to *p* < 0.05. Bold and italicized values are significant. * with bracket denotes significant difference between respective groups. # main effect of FOLFOX, $ main effect of (10 min/d) (short-duration) exercise.

Next, we examined skeletal muscle COXIV and myokine gene expression in PBS and FOLFOX-treated mice that performed repeated bouts of short-duration (10 min/d) treadmill exercise. There was a main effect for short-duration exercise to increase COXIV mRNA expression in PBS and FOLFOX mice (Figure 5A). There were no effects of short-duration exercise to alter muscle LIF or IL-6 mRNA expression (Figures 5B,C). In PBS mice, short-duration exercise reduced myostatin mRNA expression compared to sedentary PBS (Figure 5D). FOLFOX-treated mice had higher skeletal muscle myostatin mRNA expression than PBS-sedentary and short-duration exercised mice (Figure 5D). Short-duration exercise reduced muscle myostatin mRNA expression compared to sedentary FOLFOX mice (Figure 5D). There was a main effect of short-duration exercise to reduce muscle BDNF and IL-15 mRNA expression in PBS and FOLFOX mice (Figures 5E,F). There was a main effect for FOLFOX to reduce muscle LIF mRNA expression in sedentary and short-duration exercised mice (Figure 5B). Short-duration exercise for 10 min/d in mice recovering from FOLFOX chemotherapy was sufficient to induce skeletal muscle COXIV mRNA expression, similar to long-duration exercise. Short-duration exercise reduced myostatin, BDNF, and IL-15 gene expression in PBS and FOLFOX-treated mice.

### 3.6 Association between skeletal muscle COXIV activity and myokine/exerkine gene expression in sedentary and exercised mice treated with PBS or FOLFOX

**FIGURE 6:**
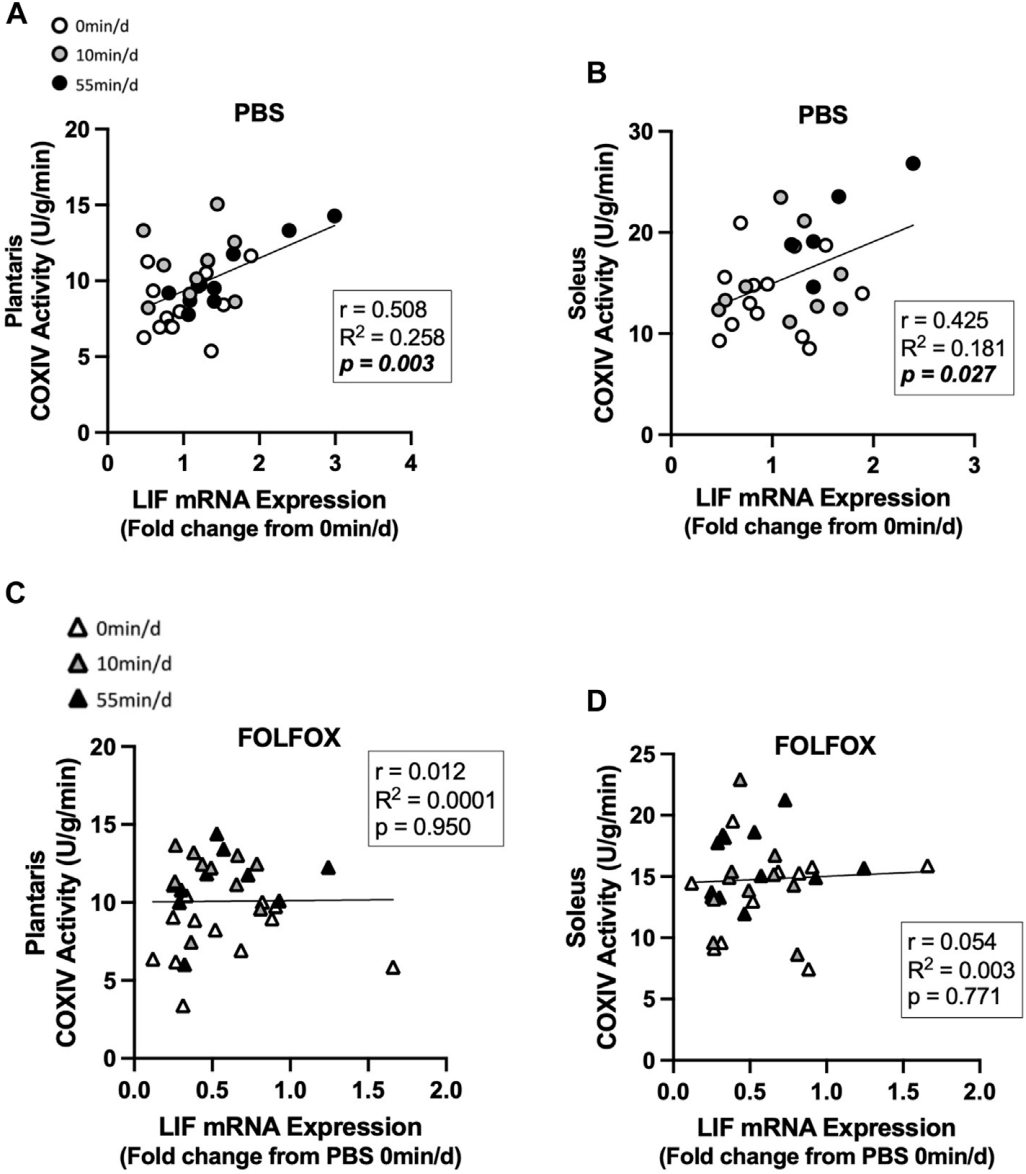
Association between skeletal muscle LIF mRNA expression and muscle COXIV activity and in sedentary and exercised PBS or FOLFOX treated mice. **(A)** Pearson r correlation of red gastrocnemius LIF mRNA expression with plantaris muscle COXIV enzyme activity and **(B)** soleus muscle COXIV enzyme activity in PBS mice. **(C)** Pearson r correlation of red gastrocnemius LIF mRNA expression with plantaris muscle COXIV enzyme activity and **(B)** soleus muscle COXIV enzyme activity in FOLFOX treated mice. Statistical significance is set to *p* < 0.05. Bold and italicized *p*-values denotes significance. Circles represent PBS treated mice, Triangles represent FOLFOX treated mice. White: 0 min/d, Grey: 10 min/d, and Black: 55 min/d.

**FIGURE 7:**
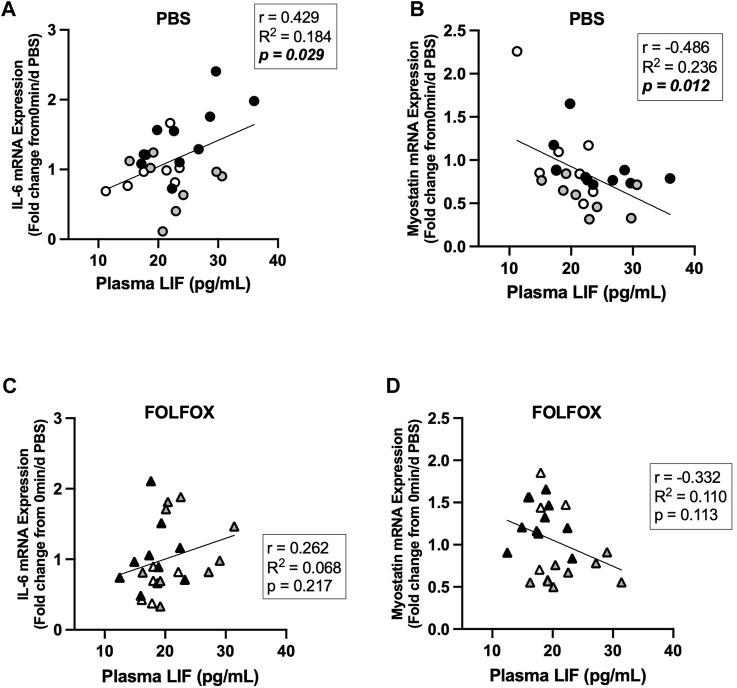
Association between plasma LIF and muscle myokine/exerkine mRNA expression in sedentary and exercised PBS or FOLFOX treated mice. **(A)** Pearson r correlation of fasting plasma LIF with red gastrocnemius muscle IL-6 mRNA expression and **(B)** myostatin mRNA expression in PBS mice. **(C)** Pearson r correlation of fasting plasma LIF with red gastrocnemius muscle IL-6 mRNA expression and **(D)** myostatin mRNA expression in FOLFOX treated mice. Statistical significance is set to *p* < 0.05. Bold and italicized *p*-values denotes significance.

Associations between skeletal muscle COXIV enzyme activity, a biomarker for oxidative metabolism-related exercise adaptations, and myokine/exerkine gene expression were examined in sedentary, short- and long-duration exercise-trained PBS and FOLFOX-treated mice. Additionally, we performed associations between systemic factors that were altered by either short- or long-duration exercise and muscle myokine/exerkine gene expression (Table 1). In PBS mice, plantaris and soleus muscle COXIV activity was positively associated with red gastrocnemius LIF mRNA expression (Figures 6A,B). Plantaris and soleus muscle COXIV enzyme activity was not associated with red gastrocnemius LIF mRNA expression in sedentary and exercised mice recovering from FOLFOX chemotherapy treatment (Figures 6C,D). Plasma LIF was positively associated with red gastrocnemius muscle IL-6 mRNA expression and negatively associated with myostatin mRNA expression in sedentary and exercise PBS mice (Figures 7A,B). There was no significant association between plasma LIF and red gastrocnemius IL-6 or myostatin mRNA expression in sedentary and exercised mice recovering from FOLFOX chemotherapy (Figures 7C,D). In sedentary and exercised PBS mice, fasting levels of plasma Glu-OCN were positively associated with red gastrocnemius muscle myostatin, BDNF, and IL-15 myokine/exerkine mRNA expression (Table 1). Fasting plasma IL-6 was positively associated with muscle IL-15 mRNA expression in sedentary and exercised PBS mice (Table 1). There were no associations between fasting plasma adiponectin and skeletal muscle myokine/exerkine gene expression in PBS mice (Table 1). Fasting plasma Glu-OCN and plasma IL-6 were negatively correlated to muscle LIF mRNA expression in sedentary and exercised mice recovering from FOLFOX (Table 1). Interestingly, plasma adiponectin was positively associated with muscle COXIV mRNA expression, both of which responded to exercise in mice recovering from FOLFOX (Table 1). These data demonstrate that muscle LIF mRNA expression is associated with skeletal muscle oxidative metabolism and mitochondrial-related adaptations in healthy exercised mice; however, prior FOLFOX chemotherapy treatment disrupts these associations.

**TABLE 1:** Values are Pearson r correlation coefficients. Group N’s PBS (N = 26–30), FOLFOX: (FOL; N = 24–30). Pearson r correlations between systemic adaptations and skeletal muscle myokine/exerkine gene expression in sedentary and exercised PBS and FOLFOX treated mice.

	Glu-OCN (ng/mL)		IL-6 (pg/mL)		Adiponectin (µg/mL)	
	PBS	FOL	PBS	FOL	PBS	FOL
***COXIV mRNA***	−0.347	−0.118	−0.062	−0.293	0.073	0.432*
***LIF mRNA***	0.121	−0.372*	0.082	−0.475*	0.027	0.064
***IL-6 mRNA***	0.180	−0.064	0.225	−0.212	<0.001	0.361
***Mstn mRNA***	0.459*	0.036	−0.001	−0.027	−0.118	−0.270
***BDNF mRNA***	0.380*	−0.159	0.342	−0.287	0.165	0.214
***IL-15 mRNA***	0.440*	0.385*	0.573*	0.050	−0.032	0.237

Abbreviations: Glu-OCN, undercarboxylated osteocalcin; IL-6, Interleukin-6; COXIV, cytochrome c oxidase complex IV; Mstn, myostatin; BDNF, brain derived neurotrophic factor; IL-15, interleukin-15; PBS, phosphate buffered saline; ng: nanograms; pg, picograms; µg, micrograms. Statistical significance is set to *p* < 0.05. Bold values with * denotes significant *p*-value.

## 4 Discussion

We have previously reported that four cycles of FOLFOX chemotherapy cause acute and long-lasting deficits in physical function and systemic metabolism in mice (Halle et al., 2022; Halle et al., 2023). Repeated sessions of exercise exert many advantageous health effects, and these benefits have been widely promoted for cancer survivors (Machado et al., 2022; Rock et al., 2022). The physiological adaptations to regular exercise can be harnessed to treat long-lasting chemotherapy toxicities that disrupt systemic metabolism and physical function after treatment. However, there is a limited mechanistic understanding of how anti-cancer treatments impact the desired exercise adaptations and whether these effects are differentially regulated based on the prescribed exercise parameters. We investigated the early adaptations to repeated bouts of short- or long-duration treadmill exercise and if prior FOLFOX chemotherapy treatment impacted the response to exercise. Associations between skeletal muscle COXIV activity, a biomarker for oxidative metabolism, and muscle myokine/exerkine-related gene expression were also examined in sedentary and exercised mice. We report the novel finding that prior FOLFOX treatment blocks the early adaptations to repeated treadmill exercise that are observed in healthy PBS mice involving circulating regulators of energy metabolism, skeletal muscle oxidative metabolism, and myokine/exerkine-related gene expression.

Regular exercise induces critical acute changes and long-term adaptations in skeletal muscle that benefit systemic metabolism and physical function (Thyfault and Bergouignan, 2020). Adaptations to exercise occur over time and can vary depending on the duration, intensity, frequency, and mode of the exercise prescribed. Skeletal muscle adaptations to exercise can also vary depending on the muscle’s functional and metabolic properties, such as slow-oxidative and fast-glycolytic skeletal muscle (Smith et al., 2023). A classic metabolic adaptation to regular treadmill exercise is increased skeletal muscle oxidative capacity involving increased mitochondria volume, biogenesis, and function (Perry and Hawley, 2018). We examined the early oxidative and glycolytic skeletal muscle adaptations to a brief, two-week training period. The effects of two physiologically relevant moderate-intensity treadmill exercise durations were investigated, relatively short 10-minutes per day, or long 55-minutes per day sessions. Exercise session length is clinically significant since it is essential to understand the critical health-related benefits from repeated bouts of shorter durations of exercise. This is especially relevant for cancer survivors suffering from fatigue and patients who may be less compliant or able to complete longer exercise sessions after the completion of cancer treatment.

Skeletal muscle COXIV activity is an indirect marker of mitochondrial oxidative metabolic capacity and has been shown to increase with regular exercise training (Booth and Narahara, 1974; Fix et al., 2018; Takahashi et al., 2022). In fact, 14 sessions of repeated aerobic exercise has been shown to increase human skeletal muscle COXIV mRNA and protein expression and citrate synthase enzyme activity (Egan et al., 2013). As expected, treadmill exercise for 55 mins per day for 14 total sessions increased oxidative soleus muscle COXIV activity by approximately 40%, while 10 mins of daily exercise did not induce a change in healthy mice. However, there was a differential response in the primarily fast-glycolytic plantaris muscle. Exercise for 10 mins a day increased plantaris COXIV activity by approximately 30%, and there was no additional benefit of exercising 55 mins per day. Interestingly, we have demonstrated that 10 mins of daily treadmill exercise during recovery from FOLFOX can improve fatigue-related functional parameters in mice and increase oxidative and glycolytic muscle mitochondria protein expression (Halle et al., 2022). We report that prior FOLFOX treatment blocked the 55 min/d induction of soleus muscle COXIV activity. However, in FOLFOX-treated mice, 10 and 55 mins of daily moderate intensity exercise for a little over 2 weeks increased plantaris muscle COXIV activity. Muscle phenotype has been reported to affect the response to many catabolic stimuli, including cancer cachexia and chemotherapy treatments (White et al., 2011; Halle et al., 2022). Further research is needed to determine if additional weeks of regular treadmill exercise could induce oxidative skeletal muscle adaptations after FOLFOX treatment. However, it is essential to point out that 14 sessions of short-duration treadmill exercise (10 min/d) effectively induced mitochondria-related adaptations in healthy and FOLFOX-treated mice. A better understanding of how repeated exercise sessions interact with chemotherapy treatment to regulate mitochondria biogenesis, dynamics, and mitochondria function will fill a critical knowledge gap on the metabolic benefits of exercise. It is intriguing to speculate that improving oxidative metabolism in fast-glycolytic muscle with relatively short bouts of treadmill exercise could benefit systemic metabolic and functional outcomes after chemotherapy.

Repeated aerobic exercise and contractile activity produce homeostatic disruptions that promote skeletal muscle mitochondria adaptations to improve endurance capacity and fatigue resistance (Perry and Hawley, 2018). Chronic contractile activity promotes ATP turnover and calcium flux, activating an array of kinases including, but not limited to, AMPK, p38MAPK, and CaMK (Hood, 2009). The exercise-induced activation of these pathways activate transcription factors that, in turn, enhance the transcription of mitochondria-related genes and translation of mitochondria-related proteins, eventually resulting in increased mitochondria volume density (Meinild Lundby et al., 2018). COXIV mRNA expression and COXIV enzyme activity were induced in response to repeated exercise bouts in healthy and FOLFOX-treated mice. The same treadmill exercise paradigm increased skeletal muscle protein expression of mitochondria OXPHOS complexes, VDAC, and COXIV in healthy and FOLFOX-treated mice (Halle et al., 2022). Indeed, exercise-induced improvements in cytochrome oxidase enzyme activity have been linked to increased mitochondrial protein content in skeletal muscle (Hollozy, 1967). Critical upstream regulators of mitochondria biogenesis can include PGC-1α and mitochondria transcription factor A (Tfam). Contractile activity has been shown to increase Tfam mRNA and protein expression, Tfam binding to mitochondrial DNA, and subsequent transcription of cytochrome c oxidase, which was associated with increased COX enzyme activity (Gordon et al., 2001). PGC-1α knockout mice exhibit reduced COX activity and blunted exercise induction of COXIV and Tfam mRNA expression (Vainshtein et al., 2015). We previously report that skeletal muscle PGC-1α protein expression was not altered by long-duration exercise; however, repeated short-duration exercise training increased glycolytic muscle PGC-1α protein expression in FOLFOX-treated mice (Halle et al., 2022), which could be linked to the observed exercise induction of COXIV in the current study. Other mechanisms underlying the exercise-induced increases in muscle COXIV enzyme activity could be related to mitophagy, whereby damaged and dysfunctional mitochondria are removed through mitochondria fission (Perry et al., 2010; Egan et al., 2013). Moreover, increased respiratory substrate availability can enhance muscle mitochondria electron transport chain enzyme activity (Reichmann et al., 1985; Henriksson et al., 1986). Further research should investigate whether chemotherapy alters exercise adaptations related to mitochondria quality control and other enzymes critical for aerobic metabolism.

Metabolic flexibility at the systemic and tissue level involves critical shifts in substrate availability and utilization to match energy demands created by feeding, fasting, and increased activity levels (Goodpaster and Sparks, 2017). Coordinated systemic substrate shifts involve crosstalk between metabolic tissues, including skeletal muscle, adipose tissue, and liver. These metabolic tissues communicate via a plethora of secreted hormones, growth factors, and cytokines. The synthesis and release of these factors are required for metabolic homeostasis during acute cell stress related to fasting, feeding, and exercise (Severinsen and Pedersen, 2020). For example, skeletal muscle secretion of IL-6 can promote fatty acid oxidation, insulin-stimulated glucose disposal, and GLUT4 translocation to the plasma membrane through activation of AMP-activated protein kinase (AMPK) (Febbraio et al., 2004; Carey et al., 2006; Kelly et al., 2009). LIF belongs to the IL-6 family of cytokines and can be induced by aerobic exercise (Broholm et al., 2008; Broholm and Pedersen, 2010). LIF can promote metabolic flexibility by promoting glucose uptake through Akt Ser 473 phosphorylation (Brandt et al., 2015). Regular exercise induces acute changes and chronic adaptations that improve systemic metabolic flexibility. Factors related to inactivity, aging, sarcopenia, and cancer cachexia have been shown to disrupt these substrate flexibility shifts and cause systemic metabolic dysfunction. We have recently demonstrated that FOLFOX chemotherapy induces acute and long-lasting systemic metabolic dysfunction in mice (Halle et al., 2023). Specifically, after 4-weeks of recovery from FOLFOX, oxygen consumption (VO2), carbon dioxide production (VCO2), energy expenditure, and carbohydrate oxidation were reduced in FOLFOX-treated mice compared to healthy controls (Halle et al., 2023). Four cycles of FOLFOX treatment acutely reduced the plasma concentration of metabolic regulators, including C-peptide, gastric inhibitor peptide (GIP), and uncarboxylated osteocalcin (Glu-OCN) (Halle et al., 2023). Many preclinical cancer cachexia models have demonstrated systemic metabolic dysfunction without chemotherapy administration (Murphy et al., 2012; Kir et al., 2014; Counts et al., 2020; Counts et al., 2021). In healthy mice, we show an exercise dose-dependent effect on the fasting response to established systemic blood plasma regulators of metabolic flexibility. Fourteen sessions of daily exercise for 55-minutes increased plasma IL-6 and LIF in healthy mice. Acute exercise-induced increases in skeletal muscle production and secretion of the IL-6 family of cytokines are well-established in humans and mice (Steensberg et al., 2000; Broholm et al., 2008; Broholm and Pedersen, 2010). However, skeletal muscle is not the only source of circulating IL-6, and this is just one of many myokines/exerkines released into circulation (Lee and Jun, 2019; Severinsen and Pedersen, 2020). Plasma IL-6 has been reported to be elevated up to 60-minutes after an acute exercise session in humans (Fonseca et al., 2021). Training-induced increases in plasma IL-6 have also been reported; mouse plasma IL-6 was elevated after 8 weeks of regular treadmill exercise (Li et al., 2021). Bone-derived Glu-OCN modulates glucose uptake, fatty acid oxidation, and can promote skeletal muscle IL-6 secretion during exercise (Mera et al., 2016). Adiponectin is a hormone secreted from adipose tissue that modulates inflammation, insulin sensitivity, and muscle energy metabolism (Yamauchi et al., 2002; Otu and Otu, 2021). However, the effects of aerobic exercise on circulating levels of adiponectin are equivocal, increasing (Kraemer et al., 2003; Oberbach et al., 2006), or not changing (Hulver et al., 2002; Marcell et al., 2005) in response to exercise. We report that moderate intensity, long-duration exercise sessions repeated for as little as 2 weeks (14 sessions) can increase fasting plasma adiponectin and Glu-OCN in healthy mice. Short-duration exercise sessions reduced plasma Glu-OCN but was insufficient to alter other systemic factors. We observed differential effects of repeated short- and long-duration exercise sessions on plasma IL-6 and Glu-OCN, suggesting that the duration of exercise per session has a critical role in the magnitude of the exercise-induction of these factors. Indeed, the duration of an exercise bout is positively associated with plasma IL-6 (Fischer, 2006). Interestingly, prior FOLFOX treatment blocked the long-duration exercise induction plasma IL-6, LIF, and Glu-OCN. The exercise induction of plasma adiponectin was maintained in FOLFOX-treated mice. Further research is needed to determine the source of the exercise-induced increases in circulating metabolic factors in response to fasting. Understanding the effects of chronic exercise training adaptations *versus* the those related to acute exercise bouts for benefiting systemic metabolic health during the recovery from FOLFOX chemotherapy requires further investigation.

Skeletal muscle cellular adaptations and responses to exercise involve a dynamic regulation of cellular signaling events from gene transcription to post-translational protein modifications during and after exercise bouts to ultimately promote long-term, whole-body adaptations (Miller et al., 2016). Muscle gene expression of IL-6 and LIF have been associated with muscle atrophy, metabolism, regeneration, and growth (Muñoz-Cánoves et al., 2013; Memme and Hood, 2021). Studies have demonstrated that aerobic exercise increases plasma IL-6 and skeletal muscle IL-6 and LIF mRNA expression immediately after exercise (Ostrowski et al., 1998; Broholm et al., 2008). The transcriptional regulation of myokines/exerkines is critical for adaptations that enhance cardiorespiratory fitness and systemic metabolism (Sabaratnam et al., 2022; Smith et al., 2023). We report that 55-minutes of moderate intensity treadmill exercise repeated daily can increase fasting muscle IL-6 mRNA expression regardless of FOLFOX chemotherapy treatment. However, in our study, skeletal muscle LIF mRNA and other myokines/exerkines were not responsive to short- or long-duration exercise. Skeletal muscle STAT3 phosphorylation was not altered by exercise despite observing exercise-related increases in upstream signaling factors IL-6 and LIF. However, it should be noted that other factors can promote skeletal muscle IL-6 gene expression, including nuclear factor-κB (NF-κB), c-Jun amino-terminal kinase (JNK), and nuclear factor of activated T cells (NFAT) (Febbraio and Pedersen, 2002). Interestingly, we report that FOLFOX chemotherapy, independent of exercise, suppresses the gene expression of myokines/exerkines, including IL-6, LIF, BDNF, and IL-15. Myostatin is a critical regulator of muscle growth, and unlike other myokines/exerkines, myostatin levels are reduced by exercise (Hittel et al., 2010; Konopka et al., 2010; Pillon et al., 2020). The 10 min/d exercise paradigm reduced muscle myostatin expression in healthy mice and was sufficient to decrease the FOLFOX-induction of muscle myostatin mRNA expression. In healthy mice, plantaris and soleus muscle COXIV activity were positively associated with muscle LIF expression. However, these associations were blocked by prior treatment with FOLFOX chemotherapy. Plasma LIF levels were positively associated with skeletal muscle IL-6 expression and negatively associated with myostatin mRNA expression in healthy mice. However, these relationships were not present in FOLFOX-treated mice. Our study suggests that both muscle gene expression and circulating levels of LIF may have important roles in oxidative metabolism adaptations to exercise, and this relationship can be impacted by FOLFOX chemotherapy. Further investigation into the metabolic role of LIF and its regulation by chemotherapy may provide critical insight into exercise health benefits after chemotherapy treatment.

Our results provide evidence that FOLFOX chemotherapy can impact early adaptations to repeated sessions of short- or long-duration exercise after the cessation of treatment. Specifically, we report the novel findings that prior FOLFOX treatment inhibits early adaptations to treadmill exercise involving circulating regulators of energy metabolism, skeletal muscle oxidative metabolism, and myokine/exerkine-related gene expression. Skeletal muscle phenotype impacted oxidative metabolism-related responses to repeated exercise after chemotherapy treatment. Specifically, the long-duration exercise induction of slow-oxidative soleus muscle COXIV activity was blunted in mice recovering from FOLFOX chemotherapy. However, the fast-glycolytic plantaris muscle responded to the short 10-minute-per-day treadmill exercise by increasing COXIV activity, and prior FOLFOX treatment had no impact on this response. Long-duration treadmill exercise significantly affected circulating metabolic regulators, including fasting plasma levels of IL-6, LIF, adiponectin, and Glu-OCN. Prior chemotherapy treatment blocked the exercise induction of fasting plasma IL-6 and Glu-OCN but did not alter the exercise induction of plasma adiponectin. FOLFOX chemotherapy had a lasting effect on fasting skeletal muscle myokine/exerkine gene expression. FOLFOX suppressed IL-6, LIF, and BDNF mRNA expression while myostatin mRNA was induced. However, skeletal muscle IL-6 mRNA expression was significantly induced by exercise in healthy and FOLFOX-treated mice. The FOLFOX chemotherapy induction of myostatin mRNA expression was significantly reduced by 10 mins of daily exercise. These differential adaptations in response to repeated exercise bouts differing in duration after FOLFOX chemotherapy treatment provide a foundational understanding that has implications for developing efficient and effective exercise prescriptions for cancer survivors.

Exercise prescription guidelines for improved physical function in cancer survivors include moderate-intensity aerobic exercise performed three times a week for at least 30-minutes per day for 12 weeks (Campbell et al., 2019). There is limited knowledge on how shorter exercise sessions performed over relatively brief time periods, compared to the 12-week programs recommended in the guidelines, can improve cancer survivor health-related outcomes soon after the completion of treatment. Our study provides evidence that important health-related exercise adaptations can occur relatively quickly, in just a couple of weeks. This finding has important clinical ramifications for cancer patients receiving immediate benefits from exercise related to metabolic health and muscle function. This is especially relevant for cancer patients and survivors who may have short windows of time to perform exercise between cancer treatments and those who may experience significant fatigue, limiting the time available to exercise in one session. Therefore, healthcare providers and rehabilitation specialists should consider using short-duration exercise sessions and expect benefits in patients after completion of several sessions.

## Data Availability

The original contributions presented in the study are included in the article/Supplementary Material, further inquiries can be directed to the corresponding author.
